# Reduced Tim-3 expression on HTLV-I Tax-specific cytotoxic T lymphocytes in HTLV-I infection

**DOI:** 10.1186/1742-4690-8-S1-A112

**Published:** 2011-06-06

**Authors:** Nashwa H Abdelbary, Hazem M Abdullah, Toshio Matsuzaki, Daisuke Hayashi, Yuetsu Tanaka, Hiroshi Takashima, Shuji Izumo, Ryuji Kubota

**Affiliations:** 1 Center for Chronic Viral Diseases, Kagoshima University, Kagoshima 890-8544, Japan; 2 Department of Neurology and Geriatrics, Kagoshima University, Kagoshima 890-8544, Japan; 3 Department of Immunology, University of the Ryukyus, Nishihara-cho, Okinawa 903-0215, Japan

## 

T-cell immunoglobulin and mucin domain-containing molecule-3 (Tim-3) and programmed cell death-1 (PD-1) are T-cell exhaustion molecules. We investigated the expression of Tim-3 and PD-1 in HTLV-I infection. Tim-3 expression, but not PD-1 expression, was reduced on CD4+ and CD8+ T cells of HAM/TSP patients and HTLV-I carriers as compared to healthy controls. Tim-3 expression was also reduced in HTLV-I Tax-specific cytotoxic T lymphocytes (CTLs) as compared to cytomegalovirus-specific CTLs. Tim-3+, but not PD-1+, Tax-specific CTLs produced less interferon-γ and exhibited low cytolytic activity. However, we observed no difference in the expression of Tim-3 or cytolytic activity between Tax-specific CTLs of HAM/TSP patients or carriers. Moreover, HTLV-I-infected CD4+ T cells showed decreased Tim-3 expression. The decreased expression of Tim-3 in HTLV-I infection is a marked contrast to other chronic viral infections such as HIV and HCV infection, where Tim-3 expression is increased in T cells, including the virus-specific CTLs. In HTLV-I infection, CTL response may not be negatively regulated by Tim-3. Rather, immune cells such as HTLV-I-specific CTLs may be resistant to cell death through the Tim-3/galectin-9 pathway. In summary, our data suggest that Tim-3 expression is reduced in HTLV-I infection and that a high number of Tim-3- HTLV-I-specific CTLs preserves their cytolytic activity, thereby controlling viral replication.

